# Effect of Water on CO_2_ Adsorption on CaNaY Zeolite: Formation of Ca^2+^(H_2_O)(CO_2_), Ca^2+^(H_2_O)(CO_2_)_2_ and Ca^2+^(H_2_O)_2_(CO_2_) Complexes

**DOI:** 10.3390/nano13162278

**Published:** 2023-08-08

**Authors:** Nikola L. Drenchev, Boris L. Shivachev, Lubomir D. Dimitrov, Konstantin I. Hadjiivanov

**Affiliations:** 1 Institute of General and Inorganic Chemistry, Bulgarian Academy of Sciences, 1113 Sofia, Bulgaria; ndrenchev@abv.bg (N.L.D.); dddimitrov_1@abv.bg (L.D.D.); 2 Institute of Mineralogy and Crystallography, Bulgarian Academy of Sciences, 1113 Sofia, Bulgaria

**Keywords:** adsorption, carbon dioxide, CaY zeolite, effect of water

## Abstract

Efficient CO_2_ capture materials must possess a high adsorption capacity, suitable CO_2_ adsorption enthalpy and resistance to water vapor. We have recently reported that Ca^2+^ cations exchanged in FAU zeolite can attach up to three CO_2_ molecules. Here we report the effect of water on the adsorption of CO_2_. Formation of Ca^2+^(H_2_O)(CO_2_), Ca^2+^(H_2_O)(CO_2_)_2_ and Ca^2+^(H_2_O)_2_(CO_2_) mixed ligand complexes were established. The Ca^2+^(H_2_O)(CO_2_) species are readily formed even at ambient temperature and are characterized by ν(^12^CO_2_) and ν(^13^CO_2_) infrared bands at 2358 and 2293 cm^−1^, respectively. The Ca^2+^(H_2_O)(CO_2_)_2_ species are produced at low temperature and are identified by a ν(^13^CO_2_) band at 2291 cm^−1^. In the presence of large amounts of water, Ca^2+^(H_2_O)_2_(CO_2_) complexes were also evidenced by ν(^12^CO_2_) and ν(^13^CO_2_) bands at 2348 and 2283 cm^−1^, respectively. The results demonstrate that, although it has a negative effect on CO_2_ adsorption uptake, water in moderate amounts does not block CO_2_ adsorption sites.

## 1. Introduction

One of the main current challenges facing society is global warming. It is mainly caused by CO_2_ emissions, and much of atmospheric carbon dioxide originates from human activity [1]. Therefore, CO_2_ capture is a very important process [2,3]. There are various ways to control CO_2_ emissions, and adsorption is considered very promising. Developed adsorbents can be conditionally divided into two main groups. One of them relies on reactive adsorption: CO_2_ is transformed into various surface species (e.g., hydrogen carbonates [4]), which then decompose during adsorbent regeneration. The second is based on a type of adsorption where CO_2_ retains its chemical identity. In this case, CO_2_ is usually bound to the surface by one or both of its oxygen atoms.

There are many works proposing the utilization of sodium-exchanged faujasite zeolites prepared from coal fly ash as CO_2_ adsorbents [5,6,7,8,9,10,11]. Indeed, to ensure a high adsorption capacity, it is necessary to use zeolites with a high concentration of exchanged cations, i.e., having a low silica-to-alumina ratio, as faujasites. However, we started our studies with NaY zeolites, because the overly high density of sodium cations in NaX has been suggested to lead to the formation of bridging CO_2_ ad-species [12,13].

We previously reported that two small molecules, such as CO and N_2_, can be simultaneously bound to one Na^+^ cation in NaY [14]. Thus, it was logical to expect that these Na^+^ sites could also be able to coordinate two CO_2_ molecules. Moreover, two CO_2_ molecules have been reported to simultaneously bind to one Na^+^ site in Na–ZSM-5 [15,16]. Indeed, in a recent study [17], we demonstrated that one Na^+^ site in the S_II_ position in NaY can attach two CO_2_ molecules. However, the second molecule binds at low temperatures or at very high CO_2_ equilibrium pressures, and therefore the process is not suitable for practical use.

To overcome this problem, we decided to change the nature of the exchanged cation. In making the selection, we considered the following factors: (i) at first, the cation should be large enough in order to be able to form geminal adsorption complexes [18,19], but not too large to significantly reduce the pore volume; (ii) the cation must be more electrophilic than Na^+^ in order to form more stable complexes with CO_2_, thereby shifting the equilibrium to higher temperatures; and (iii) the cation should be in a stable oxidation state, preferably +1 or +2 to be able to occupy a cationic position in zeolites.

Considering the possible candidates, we selected Ca^2+^ cations as the most suitable. Indeed, the cationic radius of Ca^2+^ (114 pm) is very close to that of Na^+^ (116 pm) [20]. Calcium is stable in the +2 oxidation state, and this high charge results in a high electrophilicity. Another important fact in favor of the choice of Ca^2+^ is that in CaY zeolites three CO molecules can be simultaneously coordinated to one Ca^2+^ site [21,22] (vs. two CO molecules to one Na^+^ site in NaY) [14], suggesting that a similar situation can be realized with CO_2_.

In the pilot communication to this study [23], we reported that indeed three CO_2_ molecules can be attached to one Ca^2+^ site in CaNaY. The one-ligand Ca^2+^–OCO species are produced at very low equilibrium pressure. Increasing the CO_2_ pressure leads to formation of diligand Ca^2+^(CO_2_)_2_ species, which become dominant at pressures above 1 mbar. At pressures of 65 mbar and above (or at low temperature), a third CO_2_ molecule attaches to the same Ca^2+^ site, thereby forming Ca^2+^(CO_2_)_3_ adducts.

Adsorption and desorption of CO_2_ depend on many factors: the nature and chemical composition of the adsorbent, its pore structure, temperature, pressure and the presence of other gases. Several conditions were highlighted for an effective adsorbent for CO_2_ capture [24]: high CO_2_ adsorption capacity and high CO_2_ selectivity, fast kinetics, low heat capacity, stability under extensive cycling and low-cost raw materials.

In particular, adsorption processes are usually strongly affected by water, and this is important from a practical point of view, because water is usually present in waste gases. In some cases, a small amount of water has a positive effect on CO_2_ adsorption uptake [25,26,27,28] (e.g., by creating OH groups which then act as adsorption sites [25] or by promoting the formation of hydrogen-carbonates [26,28]). However, at high concentrations, water usually suppresses CO_2_ adsorption because it concurrently occupies the adsorption sites [29,30]. Therefore, an important step in the design of CO_2_ capture materials is to study the effect of water in detail. A deep knowledge of this mechanism can help in developing strategies to minimize the negative effect of water.

In this study, we report a comprehensive picture of CO_2_ adsorption on CaNaY zeolites and how the process is affected by water vapor. In the pilot communication [23], we mainly reported the results with a CaNaY sample with a low calcium content in order to achieve an optimal intensity of the IR bands. Here we concentrate on a CaNaY sample with a high exchange degree that demonstrates a good adsorption capacity as well as examine the effect of water on the CO_2_ adsorption process. Although the main technique we used was in situ IR spectroscopy, we also utilized other complementary techniques, such as isothermal adsorption measurements, XRD, TEM with EDX elemental mapping and thermal analysis. We have found that although water has a negative effect on CO_2_ adsorption, it is mitigated due to the formation of mixed-ligand Ca^2+^(H_2_O)(CO_2_) species.

## 2. Materials and Methods

The parent NaY sample was provided by Grace Davison (SP No. 6 5257.0101, Si/Al ratio of 2.6) and was used in previous studies [14,17]. The CaNaY sample was prepared by conventional ion exchange of NaY with a 0.05 M CaCl_2_ solution (80 mL solution per 1 g zeolite). The procedure was carried out twice for 4 h at 363 K under continuous stirring. After each exchange procedure, the sample was washed extensively with deionized water and dried overnight at 393 K. Then the sample was calcined at 853 K for 5 h (heating rate 3 K min^−1^) and the described ion-exchange procedure was repeated. The sample thus prepared contained 3.96 at % Ca and 0.45 at % Na.

The FTIR spectra were recorded in transmission mode with a Thermo Scientific Nicolet 6700 FTIR spectrometer equipped with an MCT-A detector, accumulating 64 scans at a spectral resolution of 2 cm^−1^. A self-supporting pellet (ca. 10 mg cm^−2^) was prepared from the sample powder and treated directly in a purpose-made IR cell allowing measurement at ambient and low (down to ca. 100 K) temperatures. The IR cell was connected to a vacuum-adsorption apparatus with a residual pressure below 10^−3^ Pa. Prior to the adsorption experiments, the sample was activated by heating in O_2_ at 673 K followed by vacuum treatment at the same temperature. When appropriate, the spectrum of the activated sample was subtracted from the spectra registered after adsorption (background corrected spectra). A video article visualizing our experimental protocol is available [25].

CO_2_ (99.995%) was supplied by Messer. The variable temperature IR (VTIR) CO_2_ adsorption measurements were performed using a NORHOF LN2 Microdosing System #915. It allows maintaining a preset temperature between 100 and 293 K by adjusting the flow of liquid nitrogen vapor cooling the sample.

The chemical composition of the samples was determined by Energy dispersive X-ray analysis (EDX/TEM, JEOL—2100, 200 kV).

The CO_2_ adsorption isotherms were measured at 274 K using a Micromeritics 3Flex automatic surface area and pore size analyzer. Prior to the measurements, the sample was activated in vacuum at 443 or 673 K.

Thermogravimetric (TG) analyses and differential thermal analysis (DTA) were carried out on a SETSYS2400, SETARAM analyzer in the temperature range from 298 to 973 K. Al_2_O_3_ crucibles were used, and a heating rate of 10 K min^−1^ at static air was applied.

In situ HTXRD patterns of the CaNaY sample were collected using an AntonParr 600TTK XRD sample stage mounted on an Empyrean Powder X-ray diffractometer (Malvern Panalytical, The Netherlands) in the 3–90° 2θ range using Cu radiation (λ = 1.5406 Å) and a PIXcel3D detector. The powder sample was loaded in the 600TTK cuvette and dehydrated in situ at 443 K and 640 mbar for 1h (heating rate RT to 443 was 5 K min^−1^, TA Instruments degassing station). Samples were cooled to 298 K and the diffraction pattern of the dehydrated CaNaY sample was collected. Subsequently, the residual atmosphere inside the chamber was replaced by CO_2_ (avoiding contact with ambient atmosphere). After 30 min of equilibration at 298 K and 500 mbar CO_2_, the CO_2_-CaNaY pattern was collected.

## 3. Results and Discussion

### 3.1. Basic Characterization of the Samples

#### 3.1.1. Background IR Spectrum

**Figure 1:**
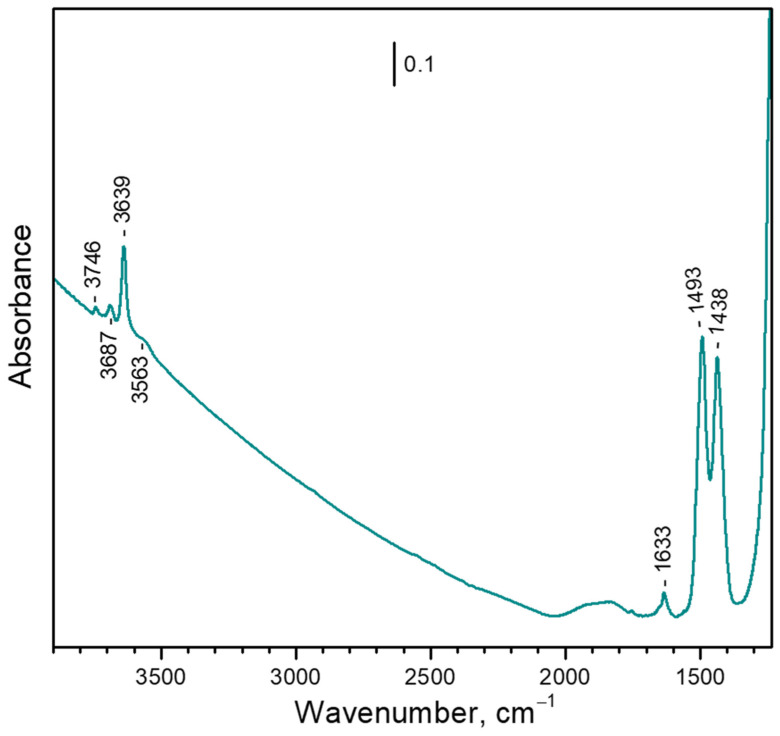
FTIR spectrum of the activated CaNaY sample.

The background IR spectrum of the sample (Figure 1, spectrum a) is typical of metal-exchanged faujasite. Zeolite skeletal vibrations are observed below 1300 cm^−1^, as well as several combination modes in the 2050–1700 cm^−1^ region. Two bands at 1493 and 1438 cm^−1^ indicate the admixture of calcium carbonate [31]. A weak feature at 1633 cm^−1^ is attributed to the δ(H_2_O) modes and shows that some water remains in the sample even after this evacuation temperature. Because the feature was practically the same after evacuation at 573 K, we infer that this residual water is occluded in some pores with blocked entrances. In the ν(OH) region, a band at 3639 cm^−1^ (absent from the spectrum of the parent NaY sample) dominates and indicates that some bridging hydroxyls were formed during the synthesis procedure [32]. A very weak band at 3746 cm^−1^ characterizes external silanols. Two bands at 3687 and 3563 cm^−1^ together with the feature at 1633 cm^−1^ are associated with the occluded water.

#### 3.1.2. X-ray Diffraction

**Figure 2:**
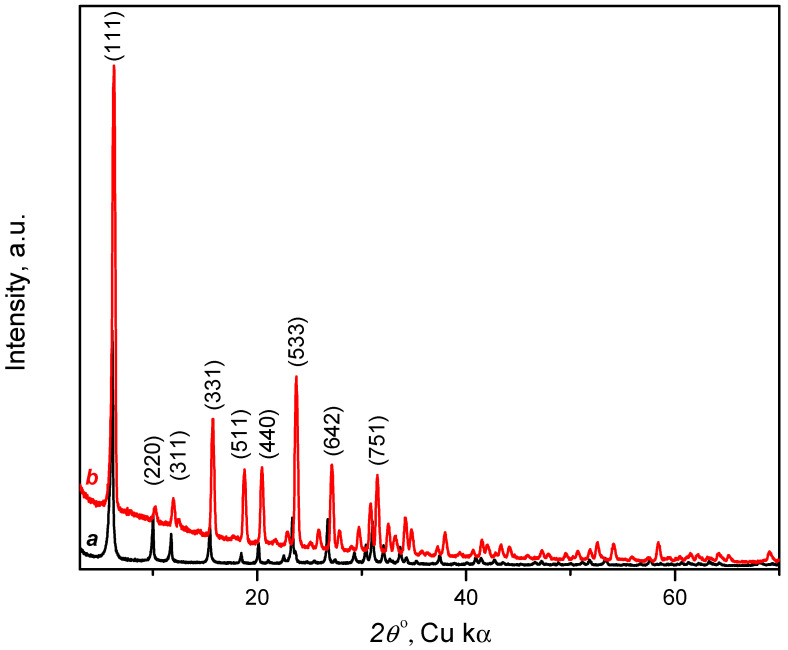
Powder diffractograms for CaNaY. Dehydrated sample (a) and in the presence of 500 mbar of CO_2_ (b).

The diffraction patterns of the CaNaY sample dehydrated at 443 K are shown in Figure 2. The pattern is in agreement with published results on dehydrated CaY zeolite [33], which confirms that the faujasite structure has been preserved after the ion-exchange procedure.

#### 3.1.3. Electron Microscopy and EDX

According to the EDX analysis, the distribution of Si, Al, Na and Ca for our sample is uniform for all these elements. The results also revealed that the Si-to-Al molar ratio (2.65) is very close to that reported for the parent NaY zeolite (2.6) (see Table S1 from the Supporting information). The (2Ca + Na)/Al ratio (0.92) is slightly lower than unity, which is justified by the presence of some acidic hydroxyls (IR band at 3639 cm^−1^).

### 3.2. Adsorption of CO_2_ in Absence of Water

The general picture of CO_2_ adsorption on dehydrated CaNaY material was reported elsewhere [23]. However, in order to obtain optimal intensities of the bands, the work concerned mainly results in a sample with low-calcium content and having a large amount of residual sodium. Here we provide some details from a CaNaY sample with a higher calcium content.

#### 3.2.1. FTIR Spectra of CO_2_ Adsorbed at Ambient Temperature

**Figure 3:**
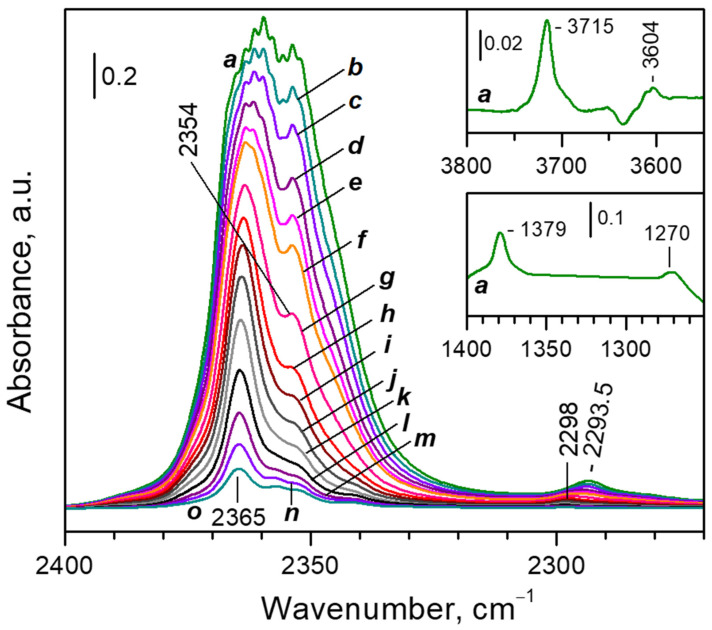
FTIR spectra of CO_2_ adsorbed on CaNaY zeolite at ambient temperature. Equilibrium CO_2_ pressure of 4 (a), 3 (b), 2 (c), 1.5 (d), 1 (e) and 0.5 mbar (f); and development of the spectra during evacuation (g–o). The spectra are background and gas-phase corrected.

Figure 3 shows the IR spectra of CO_2_ adsorbed at different equilibrium pressures on the CaNaY sample. First we will briefly describe the general picture. Because of the high calcium content, the ν_3_(^12^CO_2_) band reaches a very high intensity even at low equilibrium pressures, which leads to distortion of the spectrum [34] (Figure 3, spectra a–e). At low coverage (Figure 3, spectra g–f), a band at 2365 cm^−1^ dominates in the spectra and is associated with Ca^2+^···O=^12^C=O species formed with Ca^2+^ in S_II_ positions [23,35]. Using the value of 2339 cm^−1^ as a reference frequency for CO_2_ adsorbed in porous materials [12], the shift of the ν_3_(^12^CO_2_) modes appears to be 26 cm^−1^, which indicates a relatively strong interaction. Another band at 2354 cm^−1^ develops at slightly higher coverages. Small bands are also detected at 2357 and 2342 cm^−1^. The intensity of these bands is negligible as compared to the intensity of the ν_3_(^12^CO_2_) band at saturation. Taking this into account, along with the relatively high stability of the bands, we tentatively assign them to bridging CO_2_ (bound to the surface through its two oxygen atoms) [36].

The ν_3_(^13^CO_2_) satellite band is observed at 2298 cm^−1^ [37] and is shifted to 2293.5 cm^−1^ with coverage increase. In addition, we detected the (ν_1_ + ν_3_)(^12^CO_2_) and (2ν_2_ + ν_3_)(^12^CO_2_) combination modes [14,23,37], which at high coverage appeared at 3715 and 3604 cm^−1^, respectively (see the upper inset in Figure 3). Two more bands were recorded at lower frequencies, at 1379 and 1270 cm^−1^ (see the lower inset in Figure 3). They are attributed to the IR forbidden ν_1_ mode and the overtone of the ν_2_ vibration, respectively [14,23,37]. No other bands were detected.

**Figure 4:**
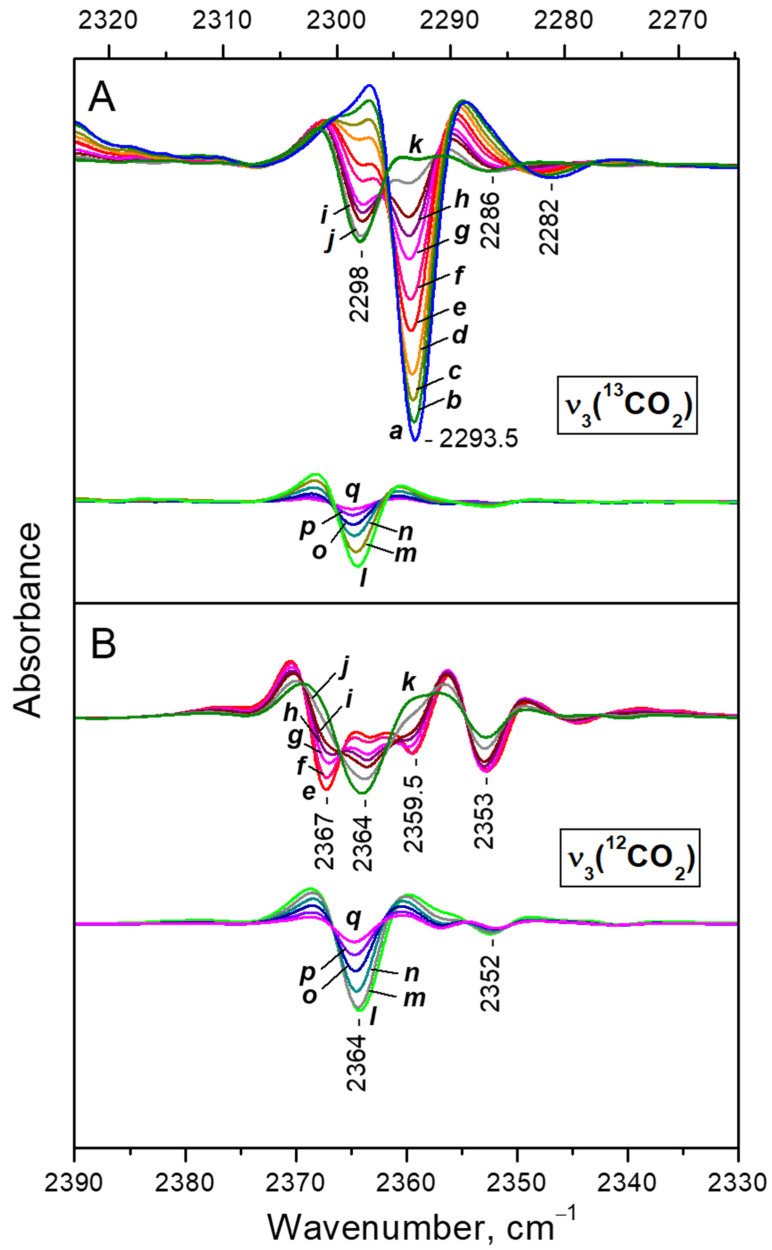
Second derivatives of the FTIR spectra of CO_2_ adsorbed at ambient temperature on CaNaY. Equilibrium CO_2_ pressure of 15 (a), 11 (b), 8 (c), 6 (d), 4 (e), 3 (f), 2 (g), 1.5 (h), 1 (i) and 0.5 mbar (j); and development of the spectra during evacuation (k–q). Panels (**A**,**B**) show the ν_3_(^13^CO_2_) and ν_3_(^12^CO_2_) regions, respectively. All spectra are background and gas-phase corrected.

In order to achieve a better resolution, for the more detailed analysis we used the second derivatives of the spectra. Figure 4 compares the ^12^CO_2_ and ^13^CO_2_ regions of the ν_3_ mode. The set of spectra in the ν_3_(^12^CO_2_) region is restricted because at high coverage the band goes out of scale. Note that the X-axis of panel B is considered with the isotopic shift factor, so the analogous bands should appear right under each other.

Consider first the ν_3_(^13^CO_2_) region (Figure 4A). Due to the low concentration of the ^13^CO_2_ isotopologue, the spectra in this region are free of any ^13^CO_2_—^13^CO_2_ vibrational interaction. For clarity we shall discuss the spectra starting from low and going to high coverage. Two main regimes of CO_2_ adsorption can be distinguished:

(i) At low coverage a band at 2298 cm^−1^ develops with the coverage increase (Figure 4A, lower set of spectra). This band is attributed to Ca^2+^···O=^13^C=O species [37].
(ii) After reaching a maximum, the 2298 cm^−1^ band starts to decline and another band at 2193 cm^−1^ develops at its expense (Figure 4A, upper set of spectra). The latter is assigned to geminal species with one labelled ligand, namely Ca^2+^(^12^CO_2_)(^13^CO_2_) [23]. Thus, the adsorption-induced shift decreases because the two CO_2_ molecules adsorbed simultaneously at the same site compete and their interaction with the cation weakens. Note that the probability of formation of Ca^2+^(^13^CO_2_)_2_ species is almost zero. Weak bands below 2290 cm^−1^ also develop. These bands are less intense as compared with a sample having higher sodium content [23] and are associated with residual Na^+^ sites.

Consider now the ν_3_(^12^CO_2_) region (Figure 4B). Here again the two adsorption regimes are well distinguished. The band developing at low coverage is located at 2364 cm^−1^ and its intensity passes through a maximum. This band was already assigned to Ca^2+^···O=^12^C=O species and the position of the ^12^CO_2_ and ^13^CO_2_ bands are in good agreement with the expected isotopic shift [38]. In this case, however, there are two main bands developing at the expense of the band at 2364 cm^−1^ when equilibrium pressure increases: at 2367 and 2353 cm^−1^. This shows that the ν_3_ mode of the geminal Ca^2+^(^12^CO_2_)_2_ species is split into two components (in-phase and out-of-phase modes) due to vibrational interaction between the two CO_2_ molecules co-adsorbed on one site [23]. The Ca^2+^(^12^CO_2_)(^13^CO_2_) mixed ligand species are expected to manifest a band around 2360 cm^−1^. Indeed, such a band was detected at 2359.5 cm^−1^. However, its intensity is higher than expected and we shall discuss this band in more detail below.

The changes of the other CO_2_ bands with the equilibrium pressure are presented in the supporting information (Figure S1 and the corresponding text).

#### 3.2.2. VTIR Experiments

**Figure 5:**
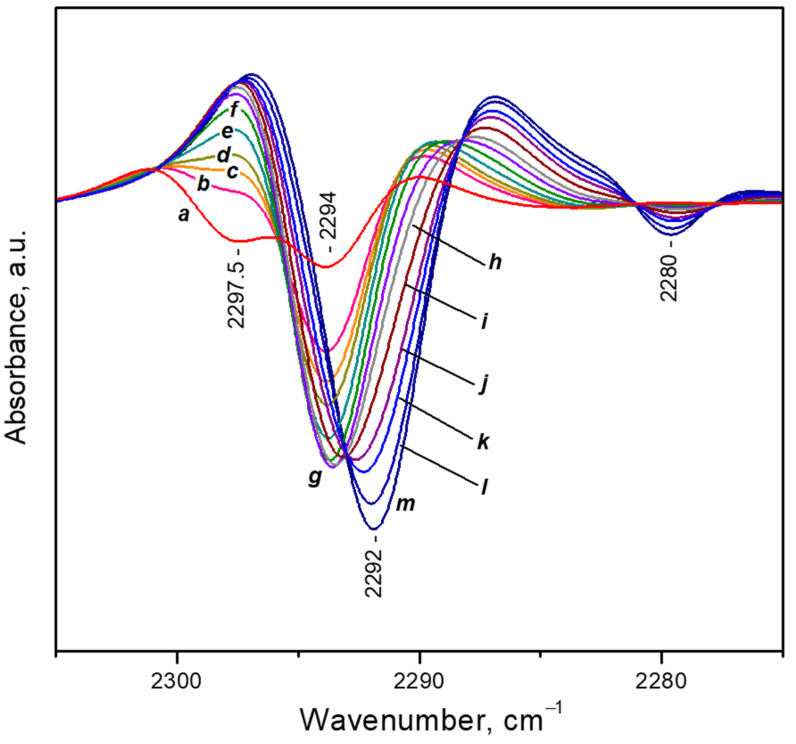
Second derivatives of the FTIR spectra of CO_2_ (initial equilibrium pressure of 2 mbar) adsorbed on CaNaY. Sample temperature of 293 K (a), 273 K (b), 263 K (c), 253 K (d), 243 K (e), 233 K (f), 223 K (g), 213 K (h), 203 K (i), 193 K (j), 183 K (k), 173 K (l) and 163 K (m). All spectra are background and gas-phase corrected.

To check for the possibility of coordination of a third CO_2_ molecule to the same Ca^2+^ site, we have performed VTIR experiments (Figure 5). They started at ambient temperature and under 2 mbar equilibrium pressure. Then the temperature was gradually decreased. At these experimental conditions it was not possible to follow the ν_3_(^12^CO_2_) band because of its very high intensity. The most intense band in the ν_3_(^13^CO_2_) region was that of diligand complexes (2294 cm^−1^), but the band of monoligand species (ca. 2298 cm^−1^) was of comparable intensity (Figure 5, spectrum a).

Decrease of temperature in the presence of CO_2_ initially leads to a fast disappearance of the 2298 cm^−1^ band and development of the band at 2294 cm^−1^ (Figure 5, spectra b–d). Further temperature lowering results in erosion of the band at 2294 cm^−1^ and development, at its expense, of a new band at 2292 cm^−1^. The latter is assigned to triligand Ca^2+^(^12^CO_2_)_2_(^13^CO_2_) species, i.e., conversion of di- to triligand complexes has occurred.

#### 3.2.3. Adsorption Isotherms

**Figure 6:**
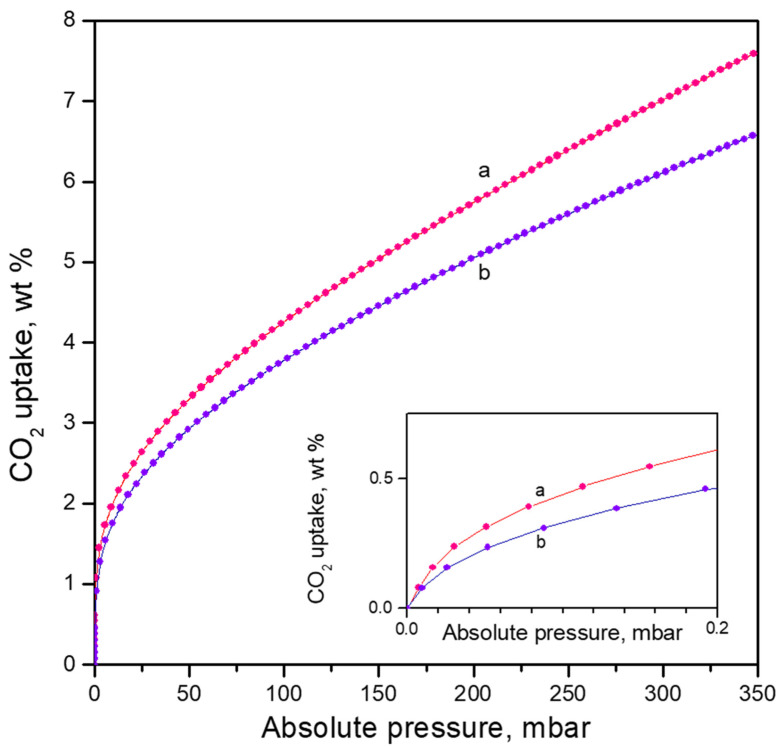
Adsorption isotherms of CO_2_ on CaNaY at 277 K. Sample activated at 673 K (a) and at 443 K (b).

The CO_2_ adsorption isotherm on the activated CaNaY sample is presented in Figure 6, curve a. The CO_2_ loading at 277 K and 350 mbar equilibrium pressure is 7.6 wt. %. The slope of the curve indicates that at these conditions the sample is far from saturation. It is also seen that a significant adsorption occurs even at very low pressures (see the inset in Figure 6).

#### 3.2.4. XRD Patterns

The diffraction pattern of the CaNaY sample in the presence of 500 mbar CO_2_ at 298 K is shown on Figure 2 (pattern b). When comparing it with the pattern of the dehydrated activated sample (pattern a), one can note variation in intensities of the peaks located at ca. 10.25, 11.95, 18.77 and 20.44° *2θ*. In addition, the shift of the peaks in the CO_2_—CaNaY pattern suggests an enlargement of the unit cell from 24.61 to 24.90 Å. At this stage attempts to establish the location of CO_2_, positioning its molecules inside the CaNaY framework using Rietveld refinement, were unsuccessful.

### 3.3. Adsorption of H_2_O on CaNaX

Although there are many studies on hydrated and dehydrated Ca-faujasites, some details on water adsorption are still unclear. For the purpose of this study, we are interested in the interaction of water with the accessible Ca^2+^ sites. It may be safely concluded that each of these sites has the capacity to coordinate up to three water molecules. Indeed, it has been established that three H_2_O-like molecules such as H_2_S [39] and NH_3_ [40] can be coordinated to one Ca^2+^ site in S_II_ position in dehydrated CaY zeolite. Moreover, it has been established that each Ca^2+^ site in the S_II_ position is coordinated to three framework oxygen atoms, having thus three coordinative vacancies allowing the acceptance of up to three guest molecules [39,40]. This is also consistent with the finding in this work that three CO_2_ molecules can be attached to one Ca^2+^ site and with earlier reports on the simultaneous coordination of three CO and N_2_ molecules [21,22]. However, the stability of the different Ca^2+^(H_2_O)_x_ (x = 1–3) species is still unknown. To obtain more information about the hydration and dehydration processes, we studied the adsorption of water and its desorption on our CaNaY sample.

#### 3.3.1. Adsorption of Water Followed by FTIR

**Figure 7:**
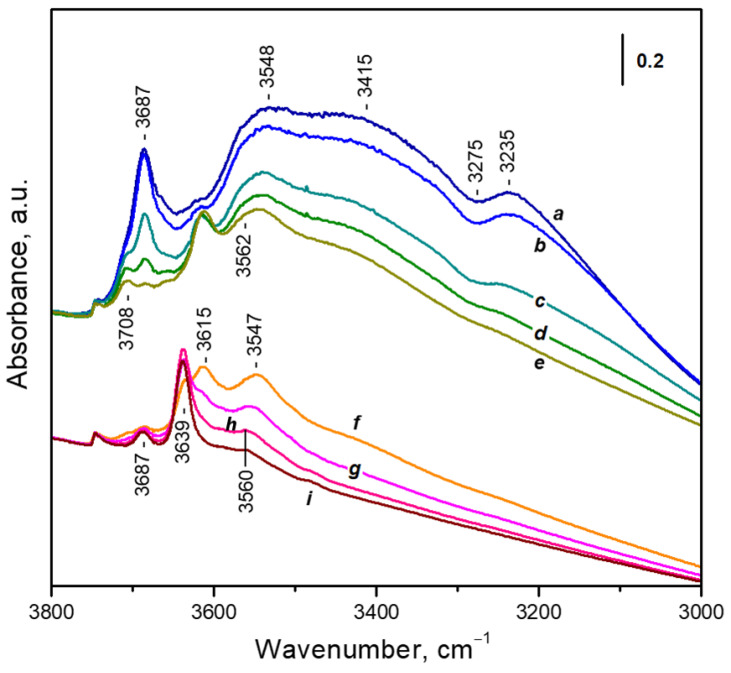
FTIR spectra of water adsorbed on CaNaY. Equilibrium H_2_O pressure of ca. 0.02 mbar (a) after 40 min evacuation at ambient temperature (b) and after 5 min evacuations at 348 K (c), 373 K (d), 423 K (e), 443 K (f), 473 K (g), 523 K (h) and 573 K (i).

The IR spectra of water adsorbed on zeolites are very complex and still there is no easy guide for their assignment. The IR spectrum of water adsorbed under equilibrium pressure of ca. 0.02 mbar is characterized by a complex δ(H_2_O) band around 1636 cm^−1^ and a more complex signal in the ν(OH) region (Figure 7, spectrum a). This signal is superimposition of the spectra of different surface species, including:

- ν(OH) modes of the acidic hydroxyl groups originally absorbing at 3639 cm^−1^ but red shifted as a result of interaction with H_2_O molecules. These are responsible for a broad band between 3600 and 3000 cm^−1^ with a typical negative feature at 3275 cm^−1^ due to Fermi resonance [32,41].
- Non-specifically adsorbed water with two ν(OH) bands, at 3687 cm^−1^ and around 3560 cm^−1^. This adsorption form also contributes to the broad absorbance between 3600 and 3000 cm^−1^ because of the formation of H-bonds [32].
- Water adsorbed on hydroxyl groups; gives rise to a band at 3708 cm^−1^ together with an ill-defined lower frequency band [42].
- A small band at 3746 cm^−1^ due to silanol groups [32].
- Unresolved bands due to water adsorbed on cationic sites. These bands will be discussed below.

Short evacuations at temperatures up to 423 K (Figure 7, spectra b–e) lead to almost full removal of the non-specifically adsorbed water, monitored by bands at 3687 and 3560 cm^−1^ and the broad feature at lower wavenumbers.

Subsequent evacuation at 443 K (Figure 7, spectrum f) results in almost full restoration of the band at 3639 cm^−1^ due to zeolite acidic hydroxyls, i.e., the respective OH–(H_2_O) adducts have been destroyed. Since Ca^2+^ ions are stronger acids than the OH groups [43], we can infer that after evacuation at this temperature only water adsorbed on cationic sites (mainly Ca^2+^ cations in S_II_ positions) remains on the surface.

Analysis of the spectra shows that the most stable band in the ν(OH) region is at 3560 cm^−1^, followed by a band at 3615 cm^−1^. We tentatively attribute these bands to complexes with one and two H_2_O molecules, respectively. Most probably the Ca^2+^(H_2_O)_3_ complexes lose one water molecule at lower temperatures.

Finally, we note that successive adsorption of small water doses on the sample leads to a slightly different picture. In this case bands due to H_2_O on OH groups and non-specifically adsorbed H_2_O are detected even after adsorption of the first doses (spectra not shown). This is due to the so-called “wall effect”, i.e., each dose of water first saturates all reached sites.

#### 3.3.2. Thermogravimetric Analysis

**Figure 8:**
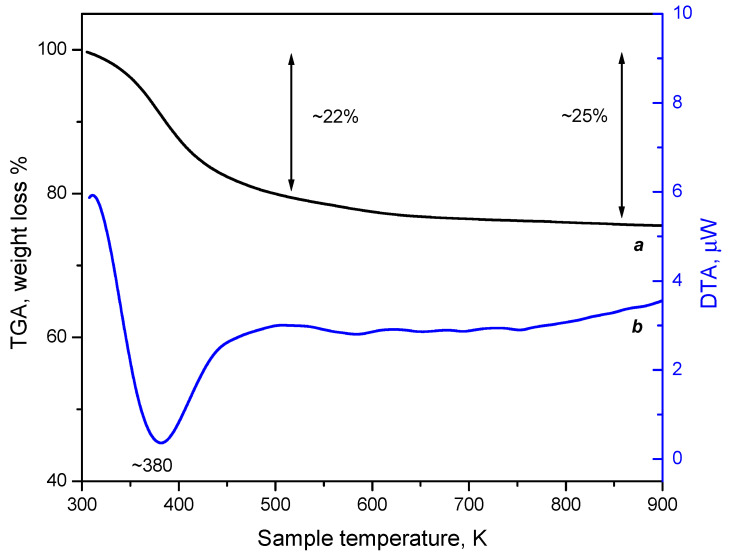
TGA (a) and DTA (b) profiles of the CaNaY sample.

The registered DTA of the CaNaY sample (Figure 8, curve a) shows a well pronounced endothermic effect starting at 300 K and finishing at ~500 K, with a maximum around 380 K. This effect is accompanied by ~22% mass loss (up to 500 K) of the sample, as registered by the TGA. The mass losses are attributed to the desorption of water from CaNaY. The TGA reveals that even after 500 K the CaNaY sample continues to lose weight, and at 900 K the mass loss is ~25%. Since the IR results have shown that the sample is not fully dehydrated after evacuation at 500 K, we may associate the mass loss above this temperature with destruction of Ca^2+^-(H_2_O) species. Indeed, the DTA curve after 500 K discloses a large but shallow endothermic effect, with a maximum at approximately ~560 K. In agreement with the FTIR results, we attribute the main peak to desorption of non-specifically sorbed water, water attached to zeolite acidic hydroxyls and destruction of Ca(H_2_O)_2,3_ species to Ca^2+^(H_2_O).

### 3.4. Co-Adsorption of CO_2_ and H_2_O

#### 3.4.1. Dosing Water on Sample with Pre-Adsorbed CO_2_ at Ambient Temperature

The next experiments were designed to establish by FTIR how water affects the adsorption of CO_2_. To this end we successively introduced small doses of water vapor to the CO_2_—CaNaY system with initial equilibrium CO_2_ pressure of 2.5 mbar. Note that the observed wall effect during water adsorption, although hindering quantification, does not affect the general conclusions because water definitely adsorbs on Ca^2+^ sites, as indicated by the IR spectra in the ν(OH) region.

**Figure 9:**
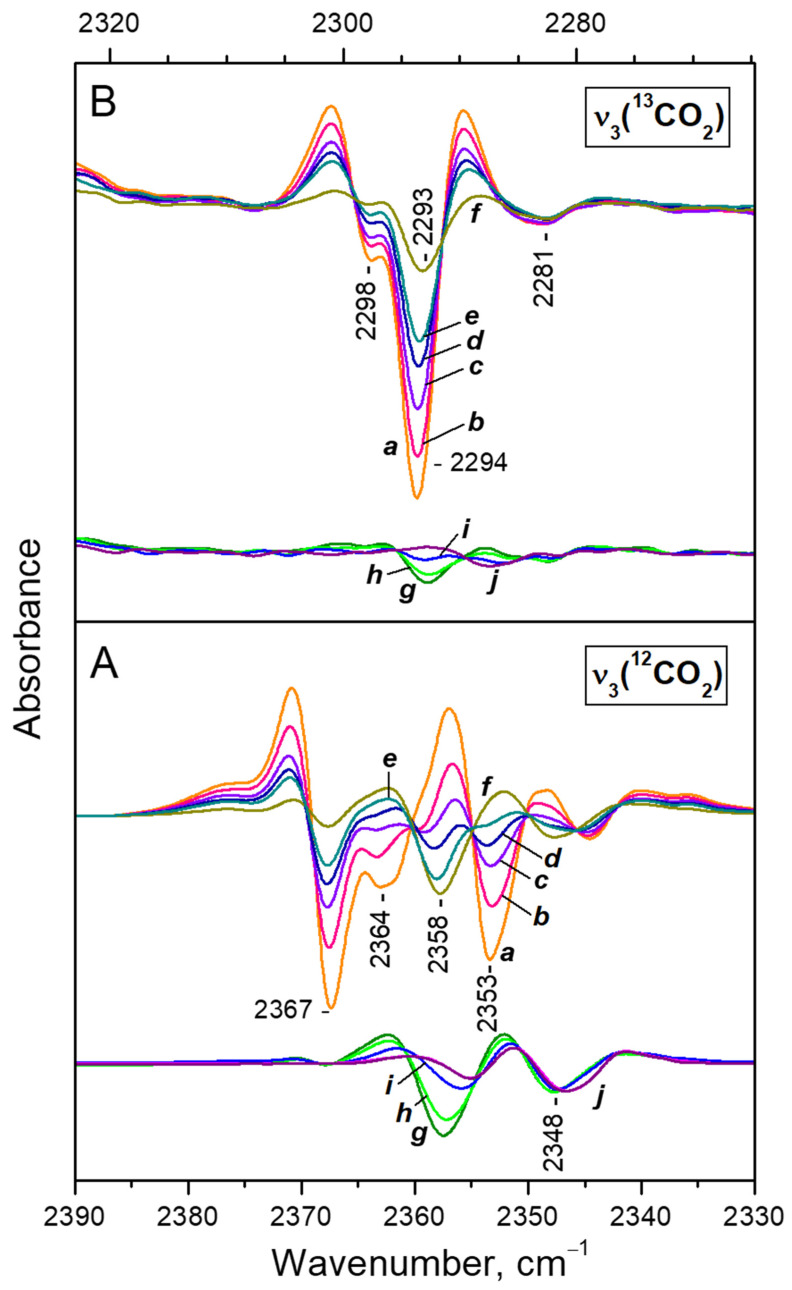
Second derivatives of the FTIR spectra of CO_2_ adsorbed at ambient temperature and equilibrium pressure of 2.5 mbar on the CaNaY sample (a) and after successive introduction of small doses of water vapor into the system (b–j). Panels (**A**,**B**) show the ν_3_(^12^CO_2_) and ν_3_(^13^CO_2_) regions, respectively. All spectra are background and gas-phase corrected.

The IR spectrum recorded before introduction of water was dominated by bands characterizing diligand Ca^2+^(CO_2_)_2_ species: 2367 and 2353 cm^−1^ in the ν_3_(^12^CO_2_) region and a band at 2293 cm^−1^ in the ν_3_(^13^CO_2_) region (see second derivatives presented in Figure 9, curve a). In addition, weaker bands due to Ca^2+^(CO_2_) adducts were also visible: ν_3_(^12^CO_2_) at 2364 cm^−1^ and ν_3_(^13^CO_2_) at 2298 cm^−1^.

Consider first the ν_3_(^12^CO_2_) region (Figure 9A). Successive dosing of water vapor into the system led to fast disappearance of the band due to linear complexes (2364 cm^−1^) and gradual decrease in intensity of the bands due to diligand species (2367 and 2353 cm^−1^). In parallel with this, a band at 2258 cm^−1^ developed. This band is at lower frequency than the band due to Ca^2+^(CO_2_) species, indicating lower electrophilicity of the adsorption site. In addition, there is no other band in the region changing in concert with it, which indicates the band characterizes monoligand species. Therefore, we assign it to mixed-ligand Ca^2+^(H_2_O)(CO_2_) adducts. Indeed, the band at 2258 cm^−1^ is at lower frequencies as compared to the Ca^2+^(CO_2_) band, which means that the CO_2_ molecule is less polarized. This is due to the fact that when a water molecule is coordinated to the Ca^2+^ site, its electrophilicity decreases. To the best of our knowledge, this is the first observation of mixed aqua-CO_2_ complexes produced by adsorption. A similar phenomenon was observed after CO and H_2_O coadsorption on Cu^+^ sites in Cu-ZSM-5, i.e., decrease in the C-O stretching frequency when a H_2_O molecule is inserted into the Cu^+^-CO complex [44].

After passing through a maximum, the intensity of the band at 2358 cm^−1^ starts to decrease and its maximum is slightly red shifted (Figure 9A, lowest set of spectra). Another band at 2348 cm^−1^ develops. Its position is close to the position of bands attributed to complexes with residual Na^+^ sites. However, the band is more intense and can thus be attributed to Ca^2+^(H_2_O)_2_(CO_2_) species. Additional arguments in favour of this assignment are provided in the next section.

Consider now in more detail the spectra in the ν_3_(^13^CO_2_) region. At first sight they seem surprising: the addition of water leads to a decrease in intensity of the bands due to mono- (2298 cm^−1^) and diligand (2294 cm^−1^) species. However, the latter band is slightly red shifted, to 2193 cm^−1^, with the increase of water coverage. Considering the isotopic shift factor, the ν_3_(^13^CO_2_) analogue of the Ca^2+^(H_2_O)(^12^CO_2_) band at 2358 cm^−1^ is expected at 2292.5 cm^−1^. Therefore, we attribute the band at 2293 cm^−1^ to Ca^2+^(H_2_O)(^13^CO_2_) adducts. This explains why the band dominates in the region when a large amount of water was adsorbed on the sample. Because of the very low intensity of the ν_3_(^13^CO_2_) bands at the end of the experiment, it was not possible to detect Ca^2+^(H_2_O)_2_(^13^CO_2_) species.

#### 3.4.2. VTIR Experiments

**Figure 10:**
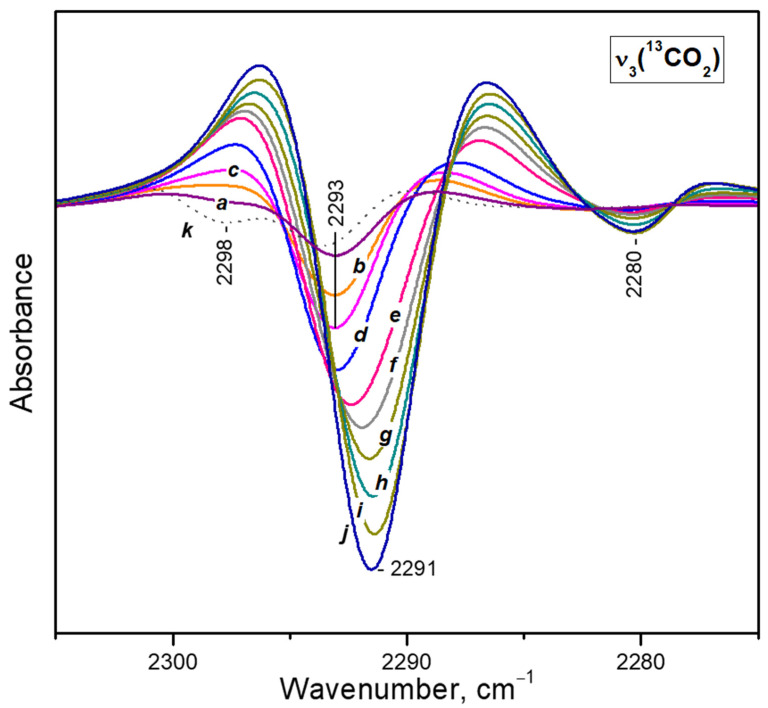
Second derivatives of the FTIR spectra of the ^13^CO_2_ bands arising from the natural ^13^C abundance (initial CO_2_ equilibrium pressure of 2 mbar) adsorbed at different temperatures on water pre-covered CaNaY (for details see text). Sample temperature of 293 K (a), 263 K (b), 253 K (c), 228 K (d), 198 K (e), 188 K (f), 180 K (g), 168 K (h), 163 K (i) and 147 K (j). Spectrum (k) is given for comparison and is registered at 293 K under 2 mbar equilibrium CO_2_ pressure with a water-free sample. All spectra are background and gas-phase corrected.

In order further to explore the coordination chemistry of calcium in the Ca^2+^(H_2_O) complexes, we performed VTIR experiments. Water was preadsorbed on the sample and then evacuated for 40 min at 443 K. After this treatment the most pronounced water bands in the ν(OH) region were at ca. 3560 cm^−1^ suggesting a large fraction of the Ca^2+^ sites are hydrated. Subsequent CO_2_ adsorption (2 mbar) produced mainly a band at 2293 cm^−1^ in the ν_3_(^13^CO_2_) region (Figure 10, spectrum a). For comparison, at these conditions a mixture of mono- and diligand species was observed with a water-free sample (Figure 10, spectrum k). Therefore, we infer that we started the experiments with a sample having mainly Ca^2+^(H_2_O)(CO_2_) complexes, whereas the fraction of water-free Ca^2+^ sites is small.

Lowering temperature leads first to an increase of the 2293 cm^−1^ band in intensity, indicating a progressive occupation of the Ca^2+^(H_2_O) sites by CO_2_ molecules. Around 200 K (Figure 10, spectrum e) the band is converted into another band located at 2291 cm^−1^. This band is attributed to Ca^2+^(H_2_O)(^12^CO_2_)(^13^CO_2_) adducts [23]. The corresponding Ca^2+^(H_2_O)(^12^CO_2_)_2_ species cannot be directly detected because of the very high intensities of the bands. The results are consistent with the previously made conclusions on the existence of three coordinative vacancies at each accessible Ca^2+^ site in our sample.

**Figure 11:**
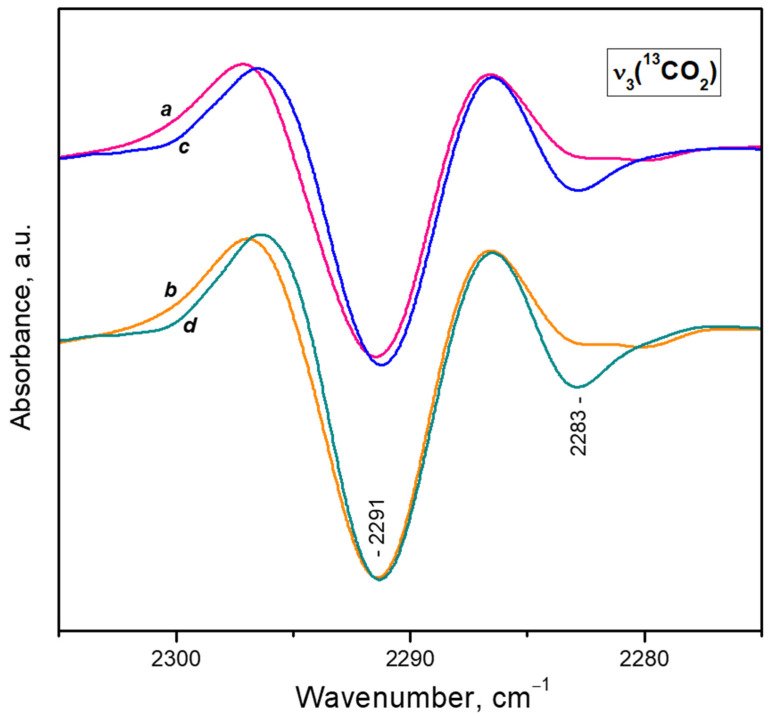
Second derivatives of the FTIR spectra of CO_2_ (initial equilibrium pressure of 2 mbar) adsorbed at different temperatures on water pre-covered CaNaY. Sample temperature of 178 K (a,c) and 168 K (b,d). Spectra (a) and (b) are registered with a sample with one additional dose of preadsorbed H_2_O and spectra (c) and (d), with two doses (for details see text). All spectra are background and gas-phase corrected.

Additional VTIR experiments were performed with samples containing larger amounts of preadsorbed water. After evacuation at 673 K, two or three doses of water (each of 10 mg per g CaY) were adsorbed on the sample, after which it was evacuated for 30 min at 373 K to remove any weakly adsorbed species. From Figure 11 it can be seen that an increase in the amount of preadsorbed water leads to a slight decrease in the integral intensity of the band at 2291 cm^−1^, characterizing Ca^2+^(H_2_O)(^12^CO_2_)(^13^CO_2_) complexes. The band at 2280 cm^−1^, associated with CO_2_ on residual Na^+^ sites, almost disappears. This is due to the occupation of Na^+^ cations (less electrophilic than Ca^2+^) by H_2_O molecules. In addition, a ^13^CO_2_ band at 2283 cm^−1^ develops. This band corresponds to the ^12^CO band at 2348 cm^−1^ already attributed to Ca^2+^(H_2_O)_2_(^12^CO_2_) species. Indeed, the formation of these species should be favored by the presence of a larger amount of preadsorbed water.

Thus, it appears that the Ca^2+^ cations occupying S_II_ positions in CaX zeolite, being coordinated to only three framework oxygen atoms, tend to reach a coordination number of six. Consequently, they can coordinate one, two or three molecules of CO and/or H_2_O, including formation of the three possible combinations with different ligands, i.e., Ca^2+^(H_2_O)(CO_2_), Ca^2+^(H_2_O)(CO_2_)_2_ and Ca^2+^(H_2_O)_2_(CO_2_).

#### 3.4.3. Adsorption Isotherms

In Figure 6 the CO_2_ adsorption isotherm on a sample activated at 443 K is compared with the isotherm obtained with a 673 K activated (dehydrated) sample. It was already discussed that after activation at 443 K a large fraction of the accessible Ca^2+^ sites are occupied by one water molecule. The results demonstrate that the adsorption capacity of the 443 K sample is lower (by 13% at 350 mbar equilibrium pressure) as compared to the dehydrated sample. This effect is more pronounced at low pressures (ca. 25% at 0.2 mbar, see the inset in Figure 6), which indicates a decrease of the initial adsorption enthalpy. This is due to the lower stability of the Ca^2+^(H_2_O)(CO_2_) complexes as compared to Ca^2+^(CO_2_).

## 4. Conclusions

Adsorption of CO_2_ at ambient temperature leads to the sequential formation of Ca^2+^(CO_2_), Ca^2+^(CO_2_)_2_ and Ca^2+^(CO_2_)_3_ complexes. The Ca^2+^(CO_2_) and Ca^2+^(CO_2_)_2_ species are formed at very low equilibrium CO_2_ pressures, and the fraction of geminal species increases with the coverage. At a pressure of ca. 65 mbar, Ca^2+^(CO_2_)_3_ species start to form. At 163 K and under ca. 2 mbar CO_2_ practically all Ca^2+^ sites coordinate three CO_2_ molecules.

In the co-presence of CO_2_ and water and at ambient temperature, Ca^2+^(H_2_O)(CO_2_) mixed ligand complexes are produced. They are in equilibrium with Ca^2+^(CO_2_), Ca^2+^(CO_2_)_2_ and Ca^2+^(H_2_O)_n_ species, which depend on the partial pressures of CO_2_ and water vapor. At low temperature Ca^2+^(H_2_O)(CO_2_)_2_ and Ca^2+^(H_2_O)_2_(CO_2_) complexes are also produced.

The results of this study show that although it has a negative effect on CO_2_ adsorption uptake, water in moderate amounts does not block the CO_2_ adsorption sites on CaNaY zeolites.

## Data Availability

The data presented in this study are available in the article and the supplementary material.

## Supplementary Materials

The following supporting information can be downloaded at: https://www.mdpi.com/article/10.3390/nano13162278/s1, Table S1: Chemical composition of the sample; Figure S1: Second derivatives of the FTIR spectra registered after adsorption of CO_2_ at ambient temperature on CaNaY (ν_1_ and 2ν_2_ regions); Explanation text to Figure S1.
