# Supplementary material for: Effect of Water on CO2 Adsorption on CaNaY Zeolite: Formation of Ca2+(H2O)(CO2), Ca2+(H2O)(CO2)2 and Ca2+(H2O)2(CO2) Complexes
Source: Nanomaterials (Basel). 2023 Aug 8;13(16):2278. doi: 10.3390/nano13162278 (PMC10458211; doi:10.3390/nano13162278)
Supplement: Supplementary file 1 [file nanomaterials-13-02278-s001.zip › nanomaterials-2526874-supplementary.pdf]

nanomaterials

Effect of water on CO_2 adsorption on CaNaY zeolite: Formation of $Ca^{2+}(H_2O)(CO_2)$, $Ca^{2+}(H_2O)(CO_2)_2$ and $Ca^{2+}(H_2O)_2(CO_2)$ complexes

Nikola L. Drenchev , Boris L. Shivachev , Lubomir D. Dimitrov and Konstantin I. Hadjiivanov

Supporting Information

Table S1. Chemical composition of the sample (at. %).

Element	Si	Al	O	Ca	Na
At. %	24.25	9.13	62.20	3.96	0.45

Si/Al atomic ratio = 2.66
Na/Al atomic ratio = 0.05
2Ca/Al atomic ratio = 0.87

FTIR spectra of CO_2 adsorbed on CaNaY (ν_1 and $2\nu_2$ regions)

The ν_1 band of CO_2 adsorbed at ambient temperature on CaNaY is located at 1378.5 cm^{-1} at low coverage and its position hardly depends on the coordination of a second CO_2 molecule: at high coverage the band is slightly shifted to 1379 cm^{-1} (Fig. S1A). In contrast, the $2\nu_2$ mode is sensitive to the formation of geminal species. At low coverage (monoligand species) the respective band is detected at 1269.5 cm^{-1} and splits into two bands, at 1274 and 1269 cm^{-1}, at high coverage, when geminal species are formed (Fig. S1B).

At low coverage the $(\nu_1+\nu_3)(^{12}CO_2)$ and $(2\nu_2+\nu_3)(^{12}CO_2)$ combination bands are detected at 3721 and 3612 cm^{-1} and are converted, when diligand species are formed, into bands at 3715.5 and 3603 cm^{-1} (spectra not shown).

When the triligand species are formed at low temperature, the $(\nu_1+\nu_3)(^{12}CO_2)$ and $(2\nu_2+\nu_3)(^{12}CO_2)$ bands are additionally red shifted and observed at 3713 and 3599 cm^{-1}, respectively (spectra not shown). The ν_1 band only increases in intensity but keeps its position. The doublet of the $2\nu_2$ band of the diligand species (1274 and 1269 cm^{-1}) is converted to a similar doublet with band maxima at 1275 and 1267 cm^{-1}.

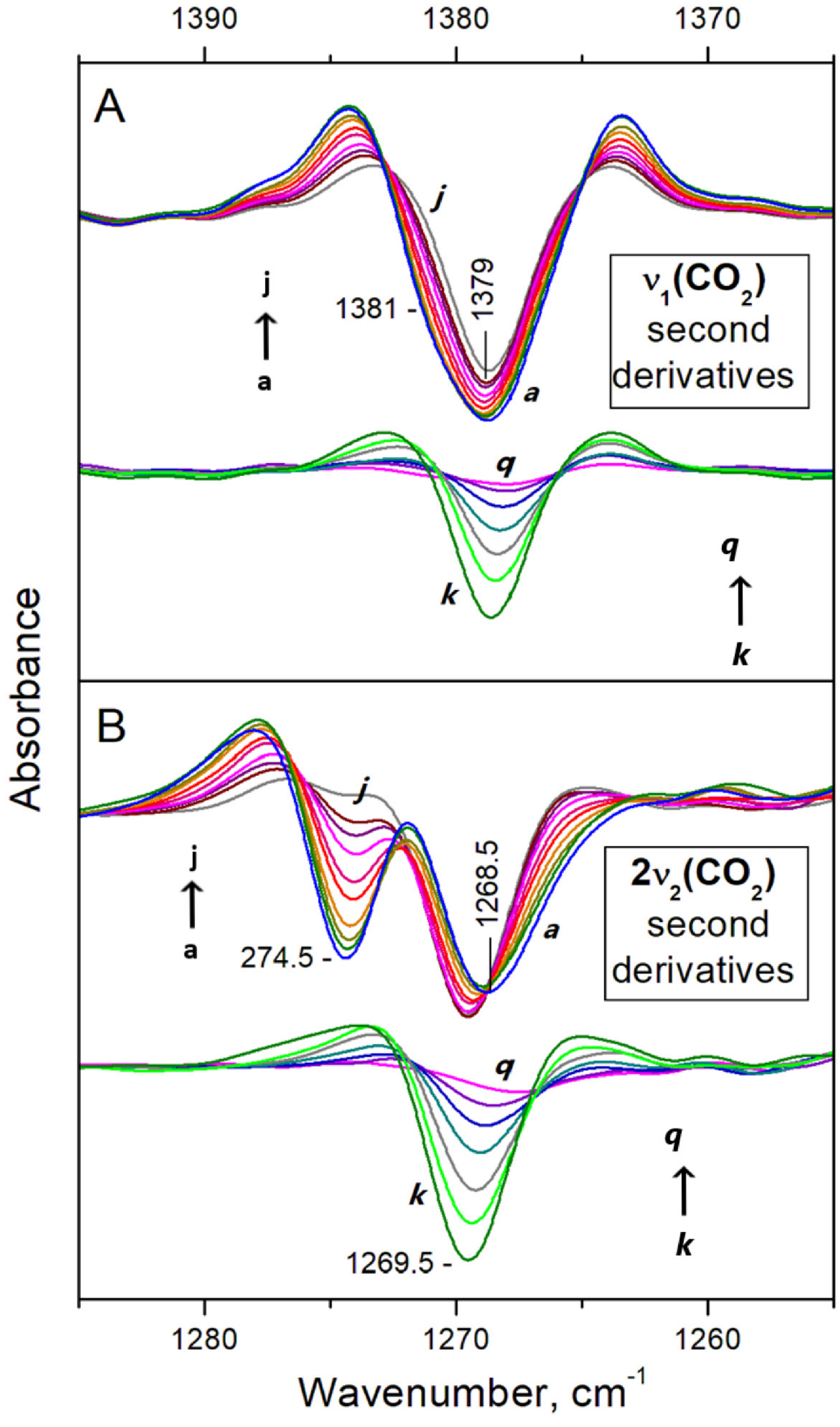

1390
1380
1370
A
j
j
a
1381 -
1379
a
ν1(CO2)
second
derivatives
q
k
q
k
Absorbance
B
j
j
a
274.5 -
1268.5
a
2ν2(CO2)
second
derivatives
q
q
k
k
1269.5 -
1280
1270
1260
Wavenumber, cm-1

Figure S1. Second derivatives of the FTIR spectra registered after adsorption of CO_2 at ambient temperature on CaNaY (ν_1 and $2\nu_2$ regions). Equilibrium CO_2 pressure of 15 (a), 11 (b), 8 (c), 6 (d), 4 (e), 3 (f), 2 (g), 1.5 (h), 1 (i) and 0.5 mbar (j) and development of the spectra during evacuation (k-g). The spectra are background and gas-phase corrected.
